# Characterization of the basic leucine zipper transcription factor family of *Neoporphyra haitanensis* and its role in acclimation to dehydration stress

**DOI:** 10.1186/s12870-023-04636-7

**Published:** 2023-12-05

**Authors:** Li Wang, Zhaolan Mo, Xinzi Yu, Yunxiang Mao

**Affiliations:** 1 Key Laboratory of Marine Genetics and Breeding (Ministry of Education), College of Marine Life Sciences, Ocean University of China, Qingdao, 266003 China; 2 Key Laboratory of Biotechnology and Bioresources Utilization (Ministry of Education), Institute of Plant Resources, Dalian Minzu University, Dalian, 116600 China; 3 Yazhou Bay Innovation Institute & Key Laboratory of Utilization and Conservation of Tropical Marine Bioresource (Ministry of Education), College of Fisheries and Life Science, Hainan Tropical Ocean University, Sanya, 572022 China; 4 Laboratory of Marine Biology and Biotechnology, Laoshan Laboratory, Qingdao, 266700 China

**Keywords:** *Neoporphyra haitanensis*, *bZIP* gene family, Dehydration, Phylogenetic analysis, Expression, Co-expression analysis

## Abstract

**Background:**

*Neoporphyra haitanensis*, a major marine crop native to southern China, grows in the harsh intertidal habitats of rocky coasts. The thallus can tolerate fluctuating and extreme environmental stresses, for example, repeated desiccation/rehydration due to the turning tides. It is also a typical model system for investigating stress tolerance mechanisms in intertidal seaweed. The basic leucine zipper (bZIP) transcription factors play important roles in the regulation of plants’ responses to environmental stress stimuli. However, little information is available regarding the bZIP family in the marine crop *Nh*. *haitanensis*.

**Results:**

We identified 19 *bZIP* genes in the *Nh*. *haitanensis* genome and described their conserved domains. Based on phylogenetic analysis, these 19 *NhhbZIP* genes, distributed unevenly on the 11 superscaffolds, were divided into four groups. In each group, there were analogous exon/intron numbers and motif compositions, along with diverse exon lengths. Cross-species collinearity analysis indicated that 17 and 9 *NhhbZIP* genes were orthologous to *bZIP* genes in *Neopyropia yezoensis* and *Porphyra umbilicalis*, respectively. Evidence from RNA sequencing (RNA-seq) data showed that the majority of *NhhbZIP* genes (73.68%) exhibited transcript abundance in all treatments. Furthermore, genes NN 2, 4 and 5 showed significantly altered expression in response to moderate dehydration, severe dehydration, and rehydration, respectively. Gene co-expression network analysis of the representative genes was carried out, followed by gene set enrichment analysis. Two *NhhbZIP* genes collectively responding to dehydration and rehydration and their co-expressing genes mainly participated in DNA repair, DNA metabolic process, and regulation of helicase activity. Two specific *NhhbZIP* genes responding to severe dehydration and their corresponding network genes were mainly involved in macromolecule modification, cellular catabolic process, and transmembrane transport. Three specific *NhhbZIP* genes responding to rehydration and their co-expression gene networks were mainly involved in the regulation of the cell cycle process and defense response.

**Conclusions:**

This study provides new insights into the structural composition, evolution, and function of the *NhhbZIP* gene family. Our results will help us to further study the functions of *bZIP* genes in response to dehydration and rehydration in *Nh*. *haitanensis* and improve *Nh*. *haitanensis* in southern China.

**Supplementary Information:**

The online version contains supplementary material available at 10.1186/s12870-023-04636-7.

## Background

Water deficit or desiccation is one of the main abiotic stresses seriously affecting plant growth and development and reducing crop production. To better survive under such harsh stress conditions, plants have evolved diverse defense mechanisms at the morphological and physiological levels driven by the regulation of stress-related gene expression [1, 2]. Transcription factors and epigenetic regulation play key roles in orchestrating gene expression and stress response when plants are exposed to extreme environmental stress conditions [3].

The basic leucine zipper (bZIP) family is one of the largest and most diverse transcription factor families [4] and is widely distributed in eukaryotes [5]. They usually possess a highly conserved 40–80 amino acid bZIP domain, which is typically composed of two structural features: a basic DNA-binding region and a leucine zipper region [5]. The basic region is highly conserved and composed of nuclear localization signals and an invariant N-X_7_-R/K motif that binds to target DNA [6], while the leucine zipper region is variable and is composed of a heptad repeat of leucine or other large hydrophobic amino acids that are involved in dimer formation in the bZIP proteins [6–8].

Several studies have shown that the *bZIP* gene family plays a vital role in many biological processes, including tissue and organ differentiation [9], metabolic activity [10], floral development [11], embryogenesis [12], and seed maturation [13]. Furthermore, bZIP proteins are also involved in the regulation of plants’ responses to abiotic and biotic stressors, such as osmotic, cold, drought, and high salinity stress. *SlAREB* overexpression in *Arabidopsis thaliana* and tomato results in enhanced tolerance to water deficit and high salinity stress [14]. In tomato, silencing *SlbZIP1* results in reduced tolerance to salt and drought stress [15]; *SlAREB1* overexpression increases salt stress tolerance [16]. Similar results have been observed for *OsbZIP23* and *OsbZIP72* in transgenic rice [17, 18] and for the *GmbZIP2* gene in soybean [19].

With the availability of whole genome sequences in various plant species, genome-wide surveys of the *bZIP* gene family have been completed in several plants. For example, 78 *bZIP* genes were found in *Arabidopsis* [8], 160 in soybean [20], 136 in *Brassica rapa* [21], and 86 in poplar [22]. However, genome-wide characterization of the *bZIP* gene family in seaweed nori has not yet been reported. *Neoporphyra haitanensis* (Bangiales, Rhodophyta), an endemic species, is an economically important marine crop that is widely cultivated along the coast of South China. At the present time, the total annual harvest of *Nh*. *haitanensis* comprises approximately 75% of all seaweed nori production in China [23]. As sessile organisms grown in harsh intertidal habitats, *Nh*. *haitanensis* is periodically exposed to air and unavoidably encounters dramatic changes in abiotic environmental conditions, such as temperature, light, desiccation/rehydration, and osmotic pressure [24–26]. This species has evolved with high adaptation to the harsh stresses of the intertidal zone habitat. *Neoporphyra haitanensis* is thus a typical model system for studying stress tolerance mechanisms in intertidal seaweed. Some progress have been achieved regarding the response of *Nh*. *haitanensis* blades to various abiotic stresses, and numerous genes, proteins, and metabolites have been identified using a single omics or multi-omics approach [26–29]. However, little information is available regarding the *bZIP* gene family in *Nh*. *haitanensis*. Recently, the genome sequences of *Nh*. *haitanensis* have been reported [30, 31], providing an opportunity to study the characteristics, evolution, and expression of the *bZIP* gene family at the genome level. Here, all members of the *bZIP* gene family were identified from the available *Nh*. *haitanensis* genome. We conducted systematic analyses of the *bZIP* genes in *Nh*. *haitanensis*, including their characteristics, phylogenetic relationships, sequence structure, pseudomolecule location, gene duplication, and synteny analysis across species. In addition, based on the RNA-seq data, the differential expression profiles of the *bZIP* genes were determined under dehydration and rehydration stress conditions. Furthermore, gene co-expression network analysis of the dehydration/rehydration responsive genes was carried out, followed by gene ontology (GO) enrichment analysis of the co-expression gene sets. Our study is the first to report on the *bZIP* gene family in *Nh*. *haitanensis*, which will provide valuable information for future studies on the function of *bZIP* genes in this important marine crop and aid in a further understanding of the molecular mechanisms underlying abiotic stress tolerance in intertidal red seaweed.

## Results

### Identification and characterization of the *bZIP* gene family in *Nh*. *haitanensis*

**Fig. 1:**
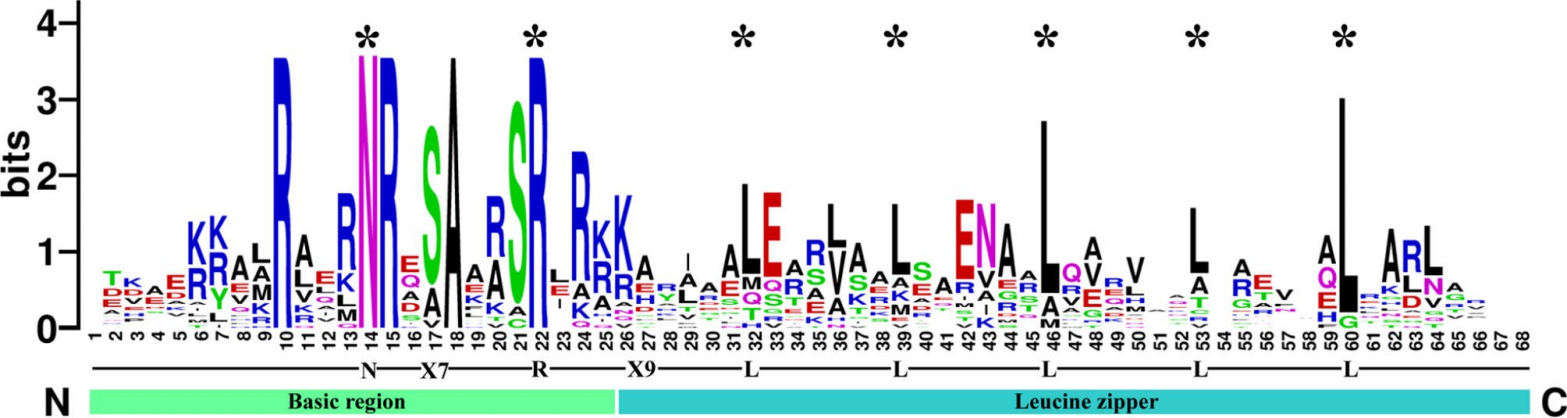
Visualization of multiple sequence alignment of the *Neoporphyra haitanensis* basic leucine zipper (NhhbZIP) transcription factor family domains. The cumulative height of the letter piles at each position demonstrates the conservation of the sequence at that position (measured in bits). The height of a single letter in the letter piles corresponds to the relative frequency of the related amino acids at that position

Initially, we identified 13 *Nh*. *haitanensis bZIP* genes using a Hidden Markov Model search (e-value < e^− 5^). We then used BLAST to search the *Nh*. *haitanensis* genome database with bZIP sequences from *Cyanidioschyzon merolae*, *Galdieria sulphuraria*, *Chlamydomonas reinhardtii*, and *A*. *thaliana* as queries. Finally, 19 *bZIP* genes were identified in the *Nh*. *haitanensis* genome, further confirmed using the SMART database (Additional file 1: Table S1), and named *NhhbZIP1*–*NhhbZIP19*. Furthermore, the amino acid sequences of the conserved bZIP domain from each member were extracted, and multiple sequence alignment was performed as described in [32]. As shown in Fig. 1, the conserved *bZIP* domain consists of a basic DNA-binding region and an adjacent leucine zipper structure. The basic DNA-binding region is composed of an invariable N-X7-R motif, whereas the leucine zipper region contains heptad repeats of leucine (L) or other hydrophobic amino acids. The highly conserved leucine residues are occasionally substituted for amino acids, including alanine, methionine, and valine. Our results are similar to those of previous research on *Arabidopsis* [6, 8].

The gene characteristics, including molecular weight (MW), coding sequence length (CDS), and isoelectric point (pI), were determined (Additional file 1: Table S1). The full length of the 19 predicted NhhbZIP proteins ranged from 63 (NhhbZIP8) to 1031 (NhhbZIP2) amino acid residues, with the CDS ranging from 192 to 3096 bp. The MW of the proteins varied from 7.26 kDa (NhhbZIP8) to 106.8 kDa (NhhbZIP2), and the pI was distributed from 4.84 (NhhbZIP13) to 11.35 (NhhbZIP8).

### Phylogenetic relationships and classification of *NhhbZIP* genes

**Fig. 2:**
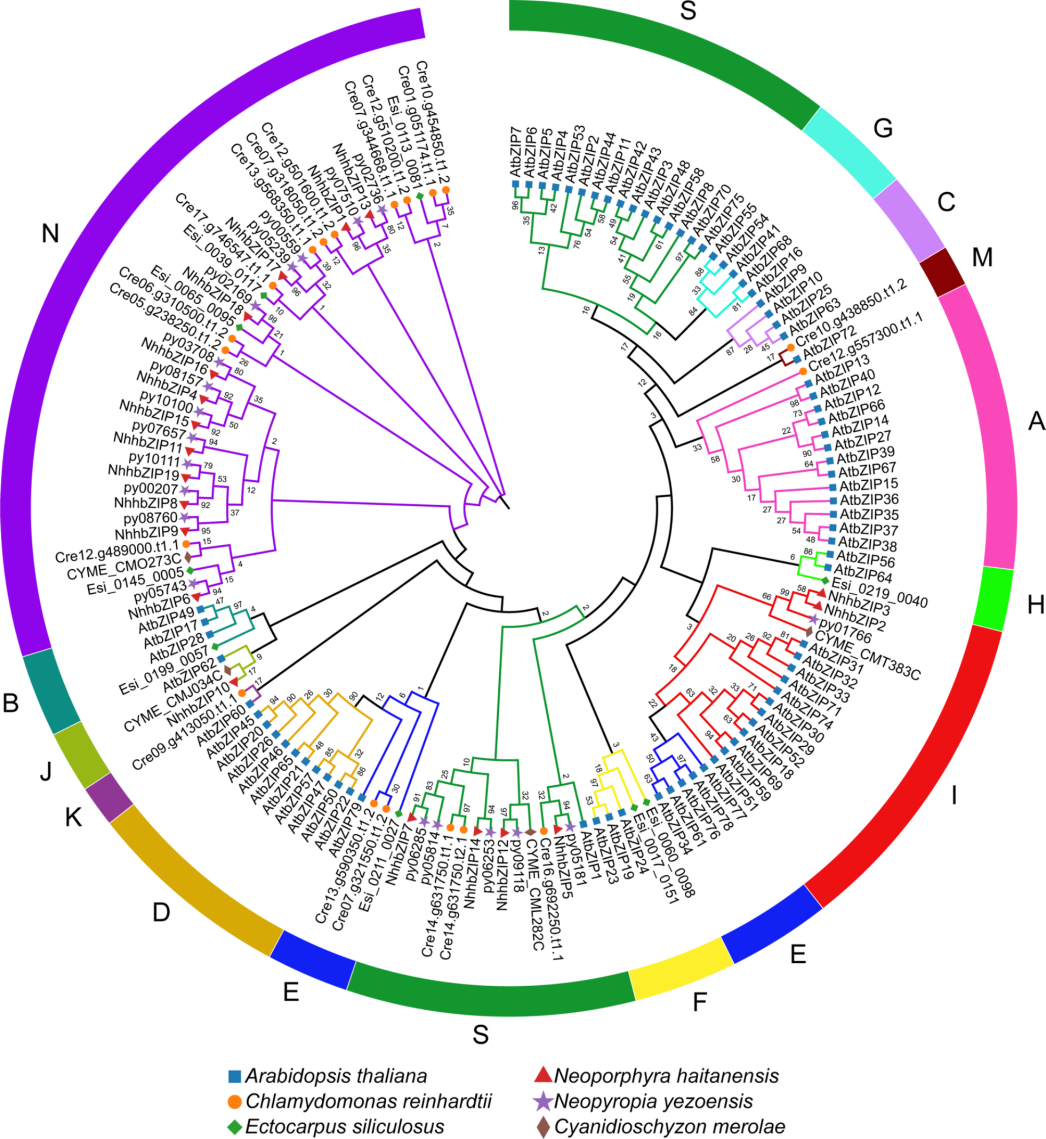
Phylogenetic analysis of the basic leucine zipper (bZIP) proteins from six different plant species (*Neoporphyra haitanensis*, *Neopyropia yezoensis*, *Cyanidioschyzon merolae*, *Ectocarpus siliculosus*, *Chlamydomonas reinhardtii*, and *Arabidopsis thaliana*). The phylogenetic tree includes 19 *bZIP* genes from *Nh*. *haitanensis*, 19 from *Ny*. *yezoensis*, 4 from *Cy*. *merolae*, 9 from *E*. *siliculosus*, 19 from *Cl*. *reinhardtii*, and 78 from *A*. *thaliana.* All *bZIP* genes from multiple species were classified into groups a, b, c, d, e, f, g, h, i, j, k, m, n, and s. Group N represents the *bZIP* genes from five algae species that cannot be classified. Different colors are used to represent different groups. The numbers beside all branches represent bootstrap values generated from 1000 replicates

To study the evolutionary relationships and classification of the *bZIP* family members, we constructed an unrooted Maximum Likelihood (ML) tree using the full-length amino acid sequences of 19 *NhhbZIPs* and five other plant *bZIPs*. As shown in Fig. 2, all *bZIP* genes were divided into 14 groups according to the classification in *A*. *thaliana* [6, 8]. Herein the *NhhbZIP* genes were separated into four groups. Group N was the biggest group, including 12 genes of the *NhhbZIP* family; whereas group J had only one member. Group N was not homologous to *A*. *thaliana bZIP* genes. Meanwhile, most *bZIP* genes of the other four algae (*Neopyropia yezoensis*, *Cy*. *merolae*, *Ectocarpus siliculosus*, and *Cl*. *reinhardtii*) were also grouped into group N, revealing that the *bZIP* gene family was relatively conserved across different algae species. These results suggest that the differentiation in the bZIP family in *Nh*. *haitanensis* and other algae is lower than that in *A*. *thaliana*.

### Sequence structure analysis of the *NhhbZIP* gene family

**Fig. 3:**
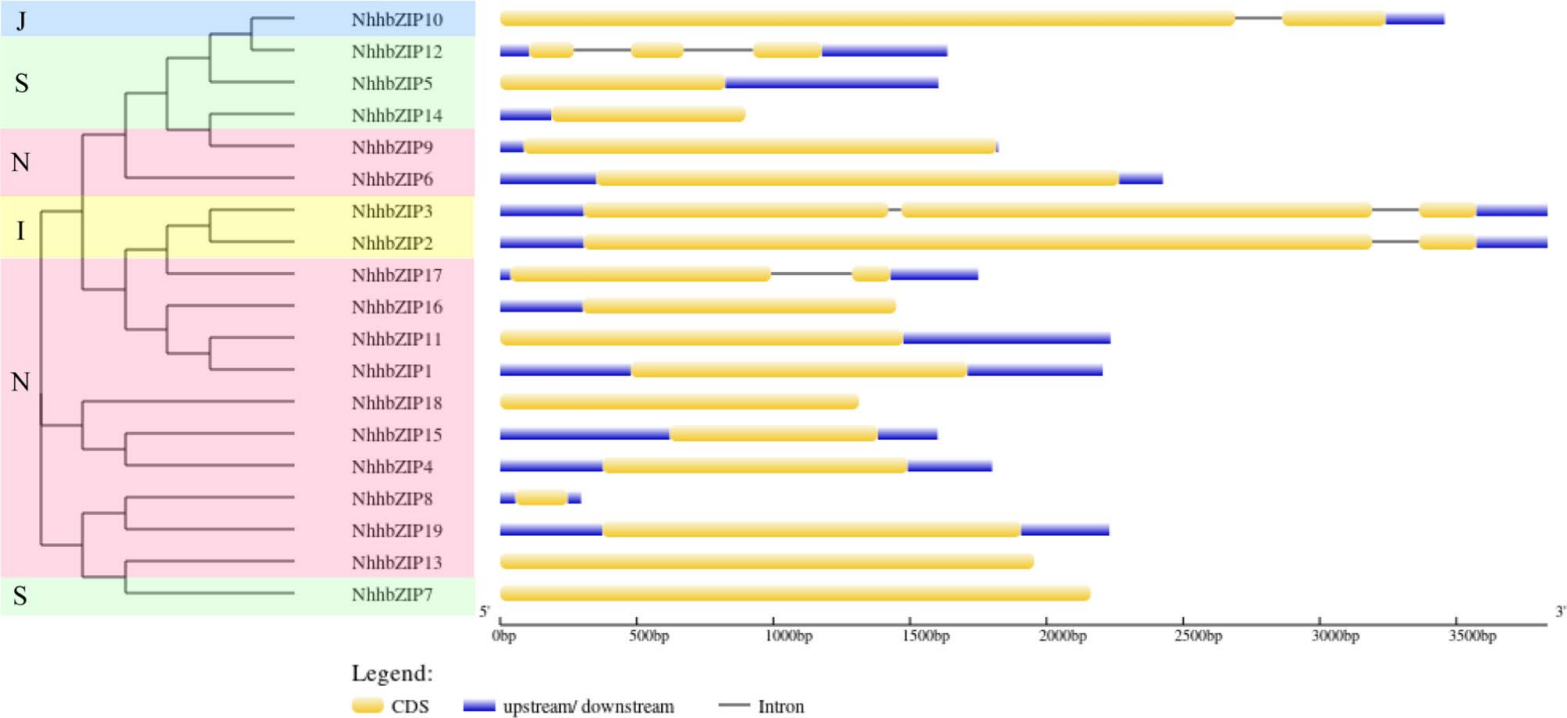
Exon–intron structures of the *Neoporphyra haitanensis* basic leucine zipper (*NhhbZIP*) genes based on evolutionary relationships. Yellow bars indicate exons; blue bars indicate 5’UTR/3’UTR; gray lines indicate introns

To study the sequence structure of the *Nh*. *haitanensis* bZIP family, we examined the exon-intron structure of each member. In general, most *NhhbZIP* genes from the same group shared a similar exon/intron number (Fig. 3). The results showed that 14 (73.7%) of 19 *NhhbZIP* genes had no introns, most of which belonged to group N. Among the intron containing genes, the intron number varied from 1 to 2. The intron number in the genes of the same group varied only slightly, mostly from 0 to 2. The number of exons showed little variation (from 1 to 3) among the different NhhbZIP groups, indicating that there were relatively smaller differences among the 19 *NhhbZIP* genes. However, the exon lengths in the genes of the same group exhibited significant variation.

**Fig. 4:**
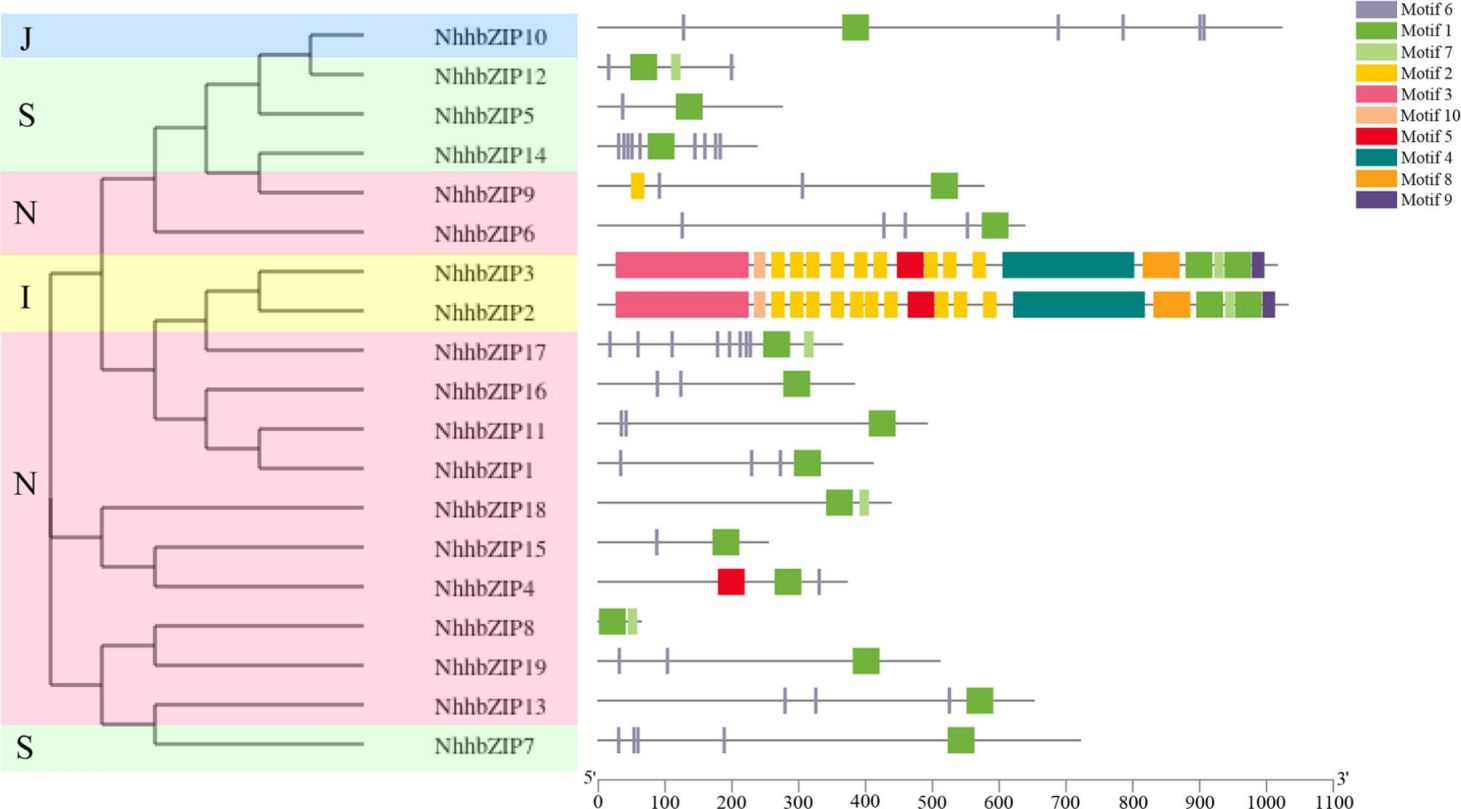
Motif compositions of the *Neoporphyra haitanensis* basic leucine zipper (NhhbZIP) proteins based on evolutionary relationships. The gray lines indicate the non-conserved sequences. The colored boxes denote different motifs

To provide insight into the divergence and function of NhhbZIP proteins, conserved motifs were predicted using MEME software. A total of 10 conserved motifs in the NhhbZIPs were identified (Fig. 4). As expected, all *Nh*. *haitanensis* bZIP members contained motif 1, which was annotated as the bZIP domain. The other motifs had no specific annotation information. Most NhhbZIP proteins within the same group had similar motif compositions. For example, group N harbored motifs 1 and 6, except for NhhbZIP8 and NhhbZIP18; all the members of group S shared motifs 1 and 6; group I possessed almost all kinds of motifs; and group J had only motifs 1 and 6. Motifs 1 and 6 widely exist in most NhhbZIP proteins. Notably, five motifs, namely motifs 3, 4, 8, 9, and 10, were found only in group I.

### Location of superscaffolds and gene duplication events of *NhhbZIP* genes

**Fig. 5:**
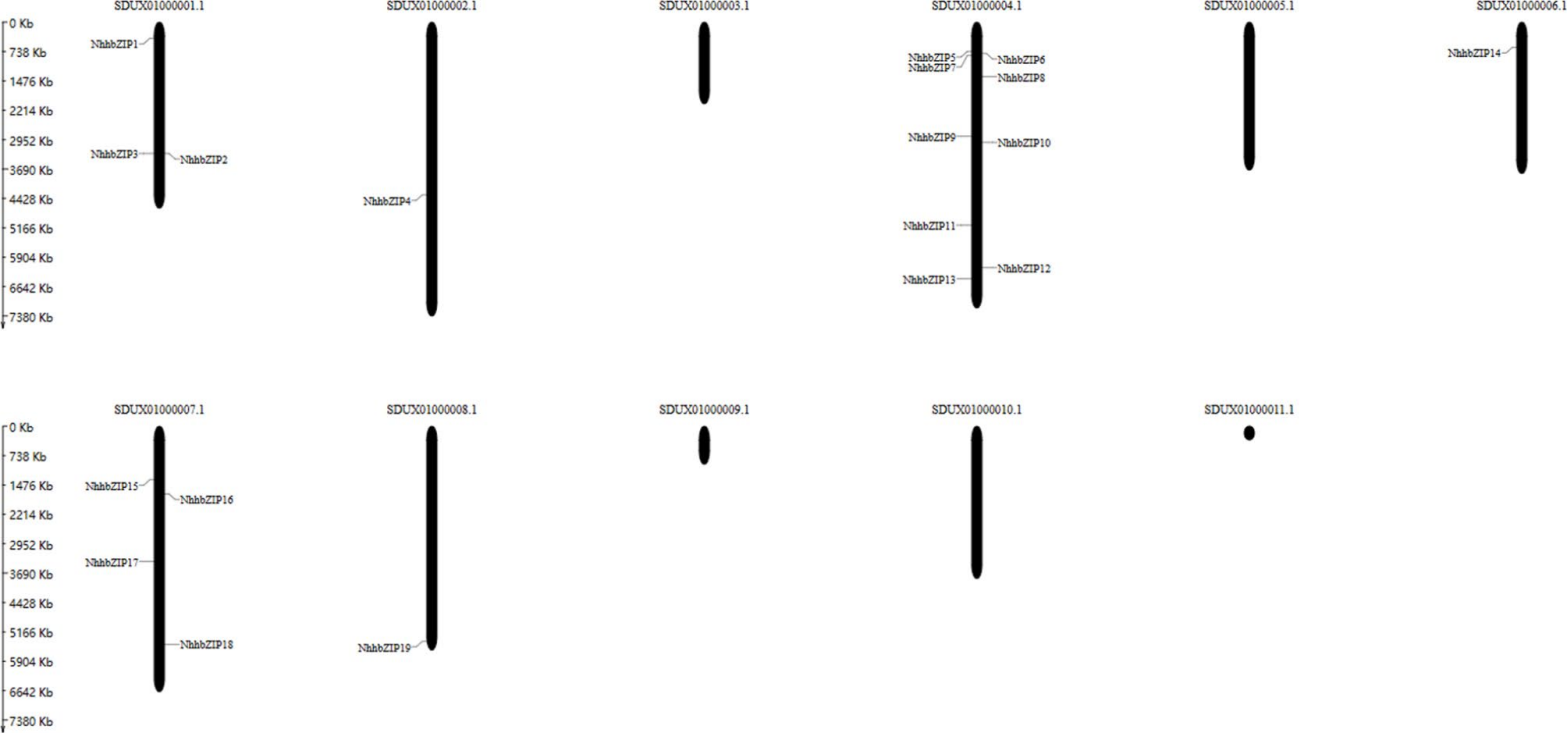
Distribution of basic leucine zipper (*NhhbZIP*) genes in *Neoporphyra haitanensis* superscaffolds. SDUX01000001.1–SDUX01000011.1 represent the 11 superscaffolds of *Nh*. *haitanensis*

As shown in Fig. 5, all 19 *NhhbZIP* genes were unevenly dispersed on the 11 superscaffolds. Specific regions showed a relatively high density of *NhhbZIP* genes. For example, SDUX01000004.1 had the most *NhhbZIP* genes (9), followed by SDUX01000007.1 with 4 and SDUX01000001.1 with 3. No genes were located on superscaffolds SDUX01000003.1, SDUX01000005.1, SDUX01000009.1, SDUX01000010.1, and SDUX01000011.1. To explore the evolutionary regulation of the *NhhbZIP* gene family, we analyzed the gene duplication events among the genes, including tandem and segmental duplication events. No tandem or segmental duplication events were found in the *NhhbZIP* gene family, revealing that the evolution of *NhhbZIP* genes was not driven by tandem or segmental duplication.

### Cross-species collinearity analysis

**Fig. 6:**
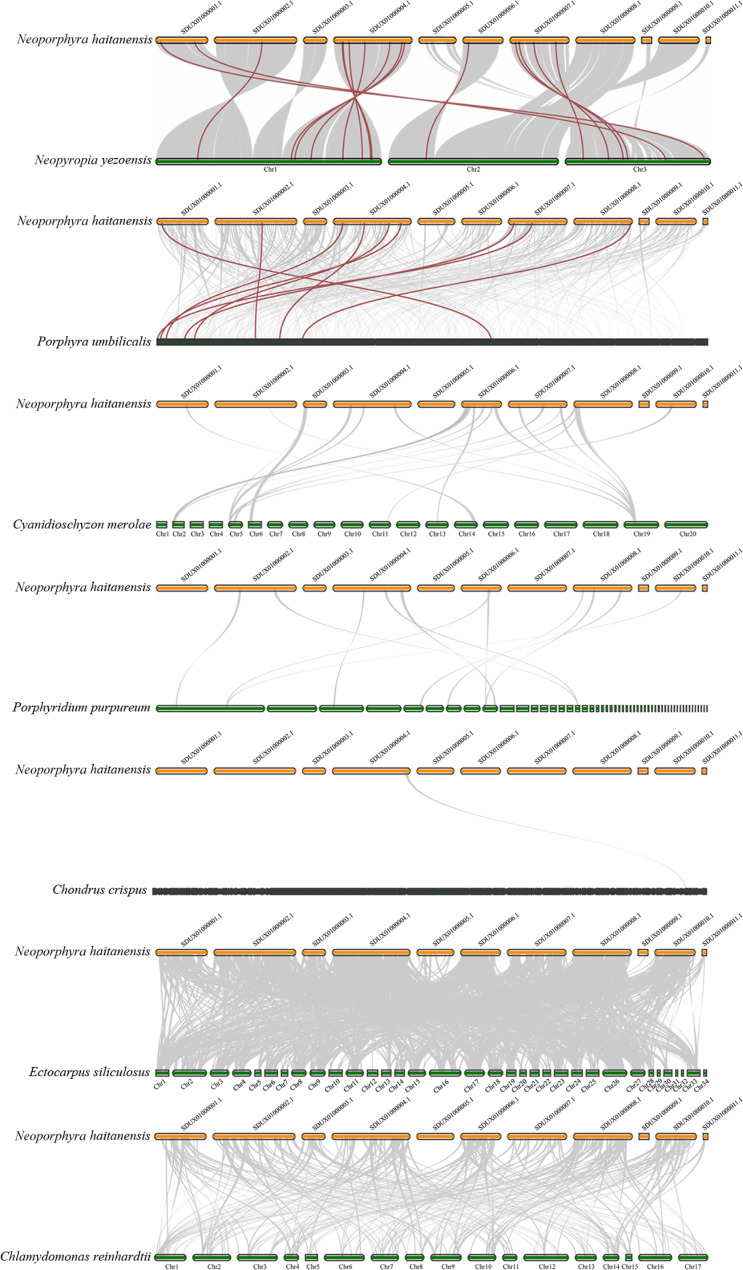
Synteny analysis of the basic leucine zipper (*bZIP*) genes between *Neoporphyra haitanensis* and seven representative algae species. The gray lines represent the collinear blocks between *Nh*. *haitanensis* and other algal genomes. The red lines represent the syntenic *bZIP* gene pairs

To analyze the collinearity relationship of the *bZIP* family genes among *Nh*. *haitanensis* and several other algae, we generated seven comparative bZIP synteny maps comparing *Nh*. *haitanensis* and seven representative algae, namely five red algae (*Cy*. *merolae*, *Chondrus crispus*, *Porphyra umbilicalis*, *Ny*. *yezoensis*, and *Porphyridium purpureum*), one brown algae (*E*. *siliculosus*), and one green algae (*Cl*. *reinhardtii*). As shown in Fig. 6, 18 *NhhbZIP* genes exhibited syntenic relationships with 16 *Ny*. *yezoensis* genes and 9 *Po*. *umbilicalis* genes (Additional file 2: Table S2). Nevertheless, no such syntenic relationship was identified between the *Nh*. *haitanensis* genes and any other algae (*Cy*. *merolae*, *E*. *siliculosus*, *Cl*. *reinhardtii*, *Ch*. *crispus*, and *Pr*. *purpureum*) genes. There were 17 pairs of orthologous genes between *Nh*. *haitanensis* and *Ny*. *yezoensis* and 9 orthologous gene pairs between *Nh*. *haitanensis* and *Po*. *umbilicalis*. These results indicate that *Nh*. *haitanensis* had a relatively closer phylogenetic relationship with *Ny*. *yezoensis* and *Po*. *umbilicalis* compared to other algae.

Notably, there was great synteny between the *Nh*. *haitanensis* genes and *Ny*. *yezoensis* genes, compared to those found in the other six algae. This is consistent with the fact that both *Nh*. *haitanensis* and *Ny*. *yezoensis* belong to *Pyropia* sensu lato. In addition, eight *Nh*. *haitanensis bZIP* genes were collectively syntenic with *Ny*. *yezoensis* and *Po*. *umbilicalis* genes. However, there was no such relationship with the genes from the other five algae, indicating that these *bZIP* genes remained in *Neoporphyra*, *Neopyropia*, and *Porphyra* and were lost in the remaining algae analyzed.

To explore the divergence of orthologous gene pairs between *Nh*. *haitanensis* and other algae, the non-synonymous (Ka)/synonymous (Ks) ratios of the syntenic gene pairs were calculated on the basis of comparative bZIP synteny maps. The Ka/Ks ratios of all orthologous gene pairs were less than 1, with the highest in the *NhhbZIP1*_*BU14_0513s0012* pair (Ka/Ks = 0.30).

### Expression profiles of *NhhbZIP* genes during dehydration and rehydration

**Fig. 7:**
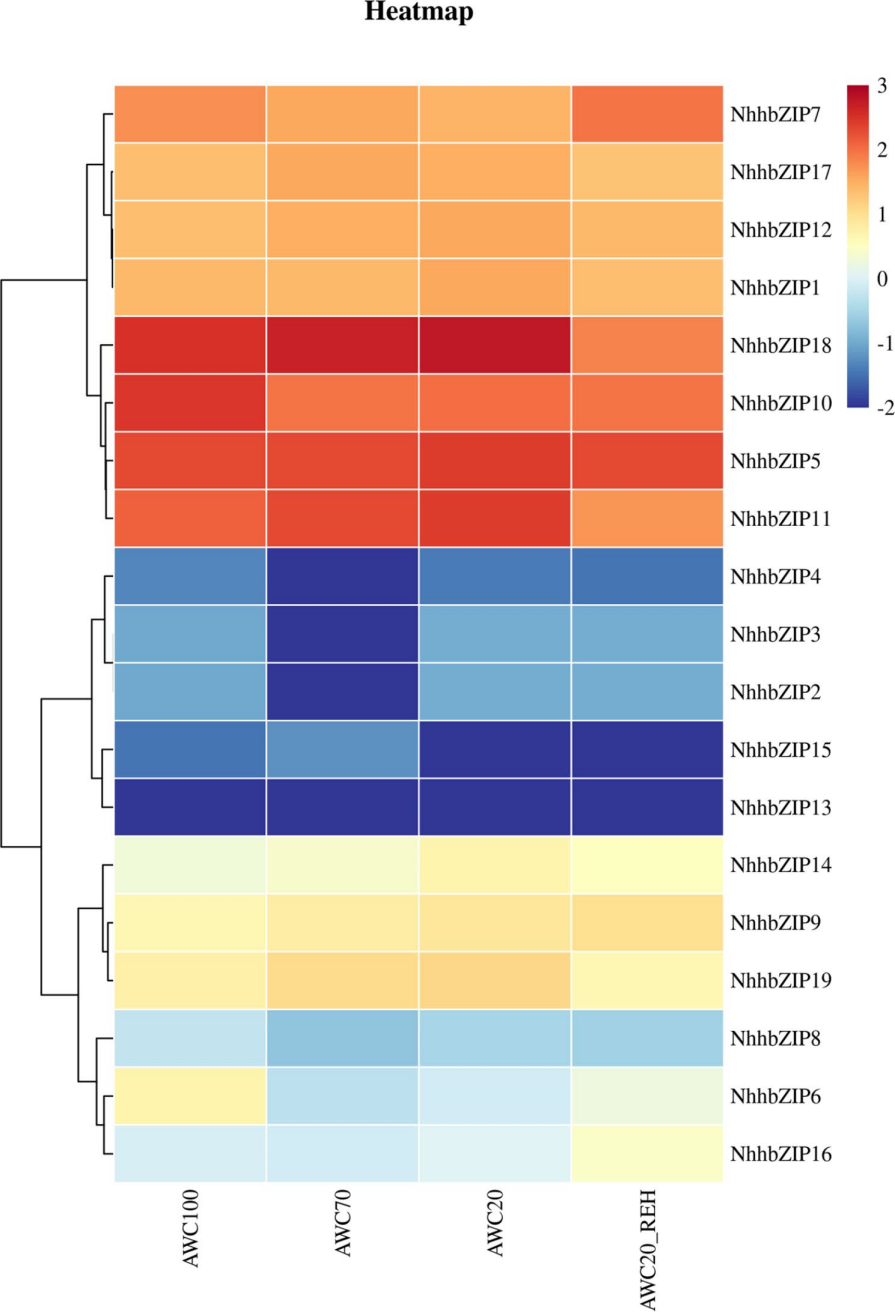
Heatmap diagram showing the expression profiles of *Neoporphyra haitanensis* basic leucine zipper (*NhhbZIP*) genes in response to dehydration and rehydration treatments. AWC100, AWC70, AWC20, and AWC20_REH represent 100% absolute water content, 70% absolute water content, 20% absolute water content, and rehydrated 30 min after 80% water content was lost, respectively. The color bar represents gene transcript abundance values normalized to log10

To investigate expression patterns of the *bZIP* genes in *Nh*. *haitanensis* in response to dehydration and rehydration, we analyzed the expression changes of these genes under dehydration and rehydration treatments, based on the RNA-seq data obtained from Wang et al. [26]. As shown in Fig. 7, five *NhhbZIP* genes had no expression or exhibited low expression in all treatments. Other *NhhbZIP* genes showed specific expression patterns in response to dehydration and rehydration treatments in *Nh*. *haitanensis*. The expression of seven *NhhbZIP* genes (*NhhbZIP1*, *NhhbZIP5*, *NhhbZIP11*, *NhhbZIP12*, *NhhbZIP14*, *NhhbZIP18*, and *NhhbZIP19*) was positively associated with the degree of water loss, i.e., the gene expression levels increased with increased dehydration and then decreased in subsequent rehydration. In contrast, one *NhhbZIP* gene (*NhhbZIP7*) showed decreased expression as the degree of dehydration deepened and then increased expression during rehydration. The expression of *NhhbZIP9* increased with increased dehydration stress, and the increase was maintained during rehydration. *NhhbZIP17* exhibited increased expression in the moderate dehydration treatment (AWC70), which then gradually decreased in the severe dehydration (AWC20) and rehydration treatment (AWC20_REH), whereas two *NhhbZIP* genes (*NhhbZIP6* and *NhhbZIP16*) showed contrasting expression patterns.

**Fig. 8:**
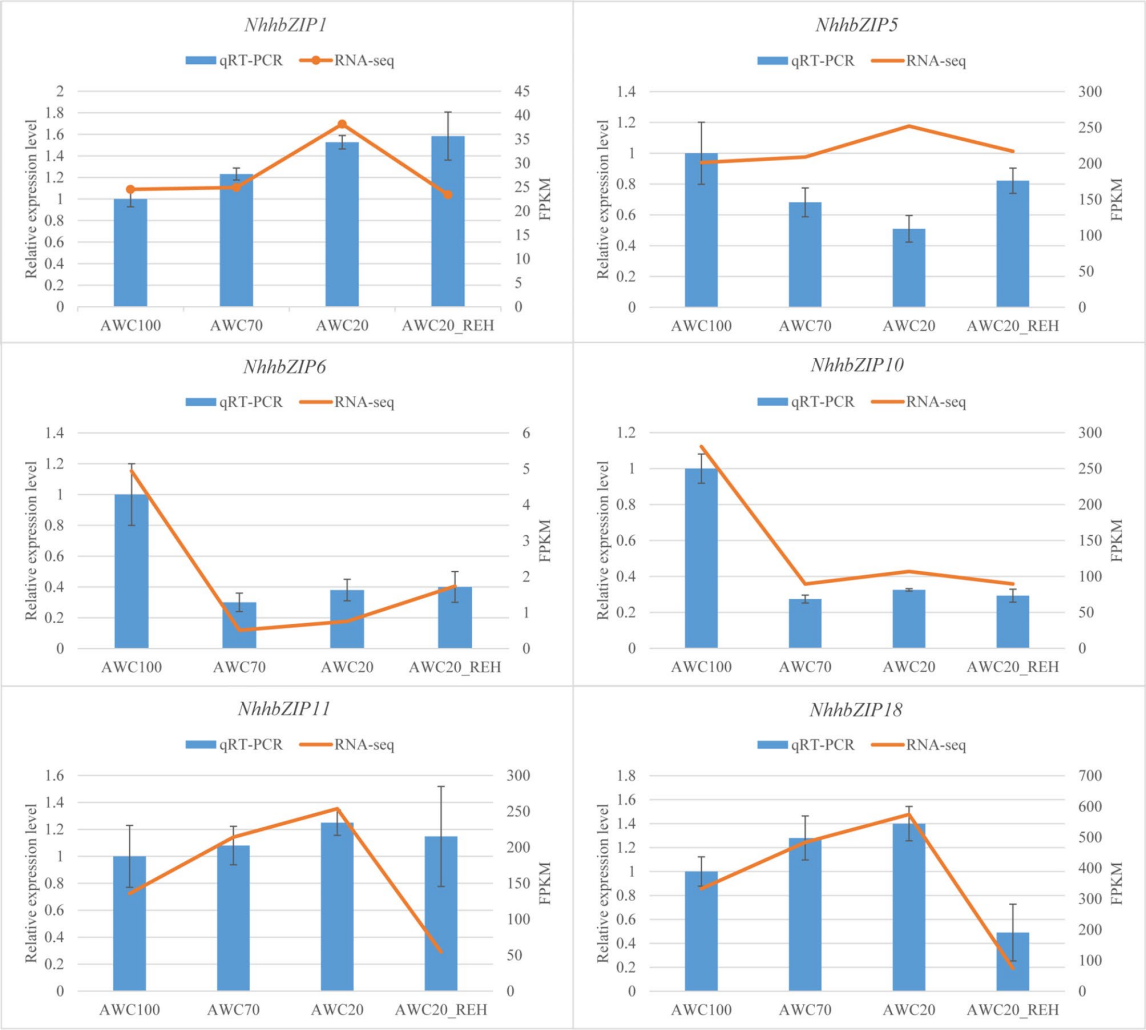
Expression patterns of six *Neoporphyra haitanensis* basic leucine zipper (*NhhbZIP*) genes under dehydration and rehydration conditions based on RNA sequencing (RNA-seq) and quantitative real-time polymerase chain reaction (qRT-PCR). The X-axis represents different stress conditions. The Y-axis represents the relative expression levels and the normalized expression data (FPKM) from the qRT-PCR analysis and RNA-seq data, respectively. The error bars indicate the standard deviations from the three biological replicates

We then used the DESeq2 method to identify differentially expressed genes (DEGs) among the various treatments. According to the results, there were two DEGs between the control group (AWC100) and AWC70 (downregulated in AWC70), followed by four genes between AWC100 and AWC20 (two downregulated and two upregulated in AWC20) and by five genes between AWC100 and AWC20_REH (four downregulated and one upregulated in AWC20_REH) (Additional file 3: Table S3). Subsequently, the three groups of DEGs were compared. As shown in Additional file 6: Fig. S1, two specific *NhhbZIP* genes were only differentially regulated between AWC100 and AWC20 (upregulated in AWC20), and three specific *NhhbZIP* genes were only involved in the response to rehydration (two downregulated and one upregulated in AWC20_REH). In addition, two *NhhbZIP* genes were collectively downregulated in the three groups of DEGs, revealing that they were collectively involved in the response to moderate dehydration, severe dehydration, and rehydration. To verify the accuracy of RNA-seq data, quantitative real-time polymerase chain reaction (qRT-PCR) analysis was used to detect the expression of six randomly selected *bZIP* genes following dehydration and rehydration treatments. As shown in Fig. 8, the qRT-PCR results were basically congruent with the RNA-seq data. However, the *bZIP* gene (*NhhbZIP5*) showed downregulated expression in response to moderate dehydration and severe dehydration and then upregulated expression in response to rehydration, which was contradictory to the RNA-seq data.

### Gene co-expression analysis

**Fig. 9:**
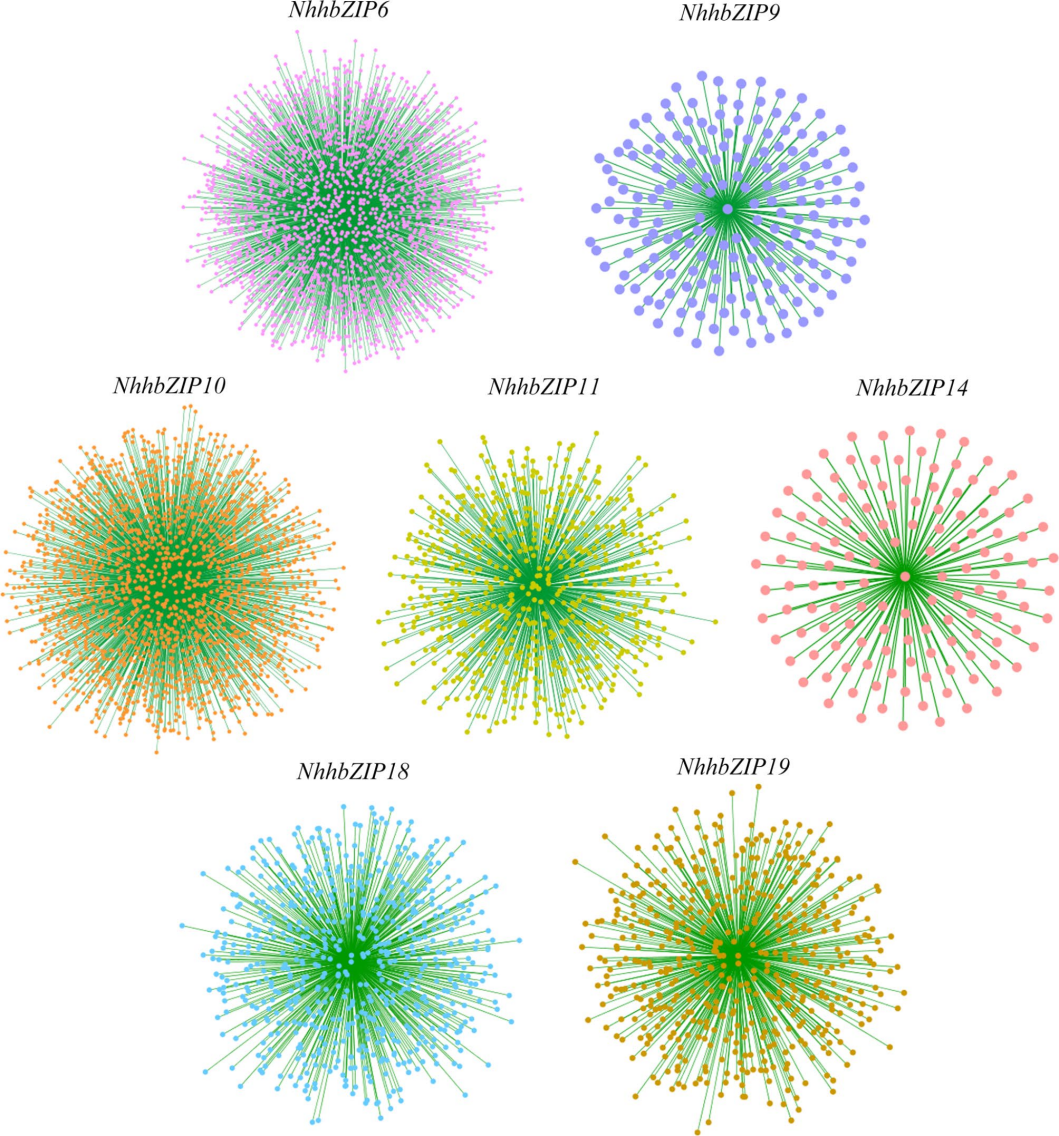
Transcription factor-focused co-expression network of two shared genes responding to dehydration and rehydration, two specific genes responding to severe dehydration, and three specific genes responding to rehydration. Dots denote genes, and lines indicate that they have a co-expression relationship

To construct a co-expression network centered on the two shared DEGs responding to dehydration and rehydration, the two specific DEGs responding to severe dehydration, and the three specific DEGs responding to rehydration, the RNA-seq data of eight samples were used for gene co-expression analysis using the Weighted Correlation Network Analysis (WGCNA) method. Seven co-expression networks were generated (Fig. 9, Additional file 4: Table S4), in which the network centering on *NhhbZIP6* was the largest (1953 genes). In contrast, the network centered on *NhhbZIP14* had the least genes (132 genes).

**Fig. 10:**
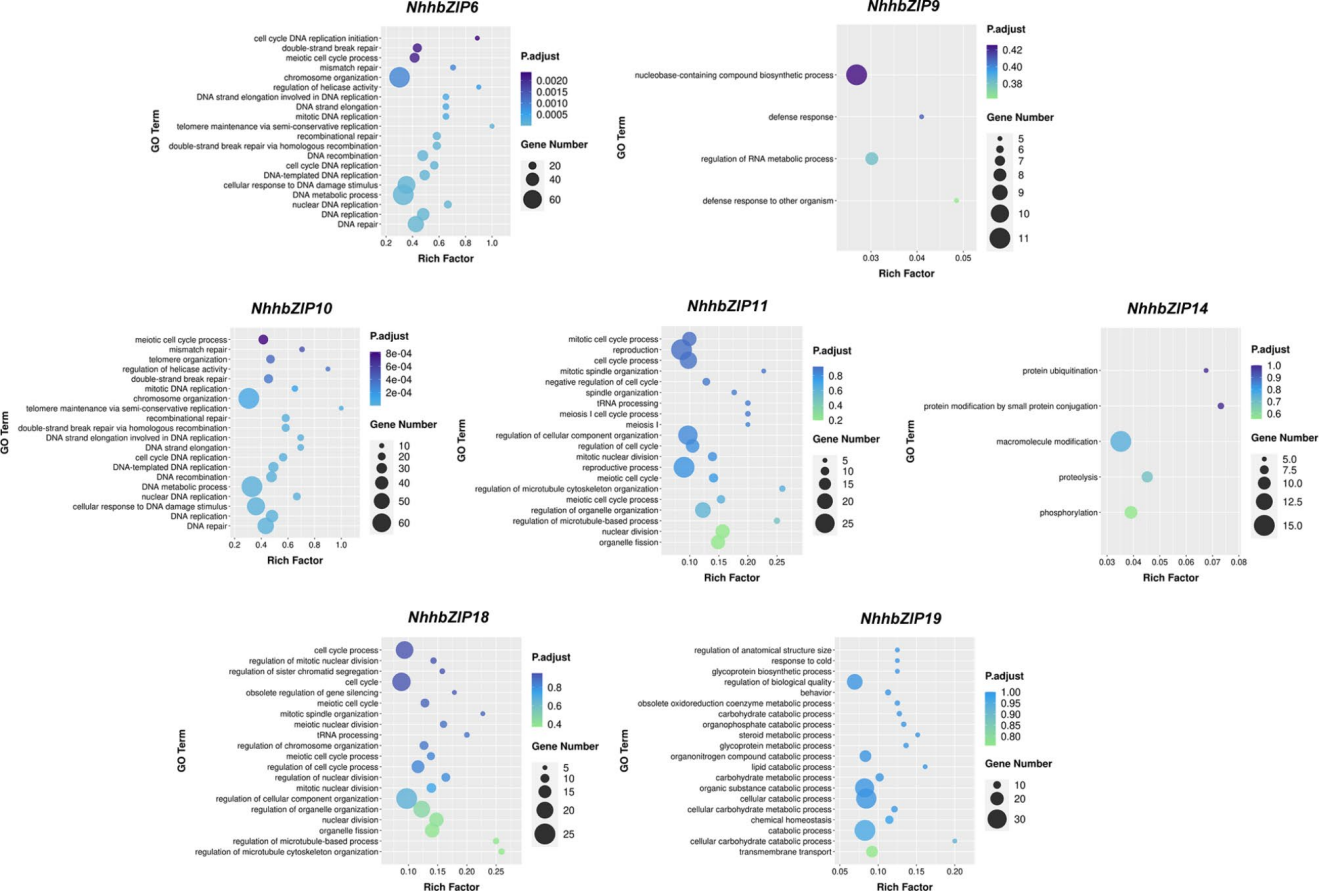
Gene ontology-based enrichment analysis of seven co-expressed gene sets

To further elucidate the biological processes in which these genes may be involved, GO enrichment tests were conducted on the seven sets of co-expression genes described above (Fig. 10). Two shared DEGs (*NhhbZIP6* and *NhhbZIP10*) share 43 significantly enriched GO terms, mainly including DNA repair, DNA replication, DNA metabolic process, cellular response to stress, cellular response to DNA damage stimulus, and regulation of helicase activity. This implies that the two genes might play crucial roles in the regulation of the cellular response to dehydration and rehydration treatments.

With respect to the two specific DEGs responding to severe dehydration, *NhhbZIP14* and the genes in its network were enriched in macromolecule modification, protein ubiquitination, proteolysis, phosphorylation, and protein modification by small protein conjugation. *NhhbZIP19* and its co-expression gene network were enriched in GO terms, including cellular catabolic process, transmembrane transport, lipid catabolic process, carbohydrate metabolic process, and other biological processes. Interestingly, the two severe dehydration-responsive genes showed the same expression pattern across the different stress conditions.

Regarding the three specific DEGs responding to rehydration, the networks centered on *NhhbZIP11* and *NhhbZIP18* shared the most enriched GO terms, such as nuclear division, organelle fission, regulation of microtubule cytoskeleton organization, regulation of cell cycle process, and reproductive process. Meanwhile, *NhhbZIP11* and *NhhbZIP18* also had the same expression profile across stress conditions. In addition, *NhhbZIP9* and its co-expression gene network were enriched in the defense response to other organisms, defense response, nucleobase-containing compound biosynthetic process, and regulation of the RNA metabolic process.

## Discussion

*Neoporphyra haitanensis*, an important marine crop native to China, thrives in the challenging habitat of the intertidal zone, and it has therefore evolved a set of protective mechanisms that make this species highly adaptable to harsh stresses. The *bZIP* gene family has been reported to participate in a variety of biological processes, such as plant growth and development and response to biotic and abiotic stress [20]. Although the *bZIP* gene family has been found in many plant species, systematic research on the *bZIP* gene family in *Nh*. *haitanensis* is relatively lacking.

Here, 19 *bZIP* family genes were identified in the *Nh*. *haitanensis* genome (53 Mb) [30]. Furthermore, 19 *bZIP* family genes were identified in the *Ny*. *yezoensis* genome (108 Mb) [33], 4 in *Cy*. *merolae* (17 Mb), 20 in *Ga*. *sulphuraria* (14 Mb), 23 in *Po*. *umbilicalis* (88 Mb), 18 in *Ch*. *crispus* (105 Mb), 18 in *Pr*. *purpureum* (22 Mb), 9 in *E*. *siliculosus* (196 Mb), and 19 in *Cl*. *reinhardtii* (111 Mb) from their genome databases and the PlnTFDB and Phytozome databases. These data suggest that there is no direct relationship between the number of *bZIP* family genes and the genome size of these algae.

Phylogenetic analysis showed that the *Nh*. *haitanensis bZIP* family could be divided into four groups based on an unrooted ML tree that compared *NhhbZIP* family members with five other plant *bZIP* genes (Fig. 2). This is less than the number of groups in most higher plants [20, 22, 34]. As described in the results, group N was not present in *A*. *thaliana*, so the majority of *bZIP* genes from five algae species could not be classified. Thus we segregated these unclassified *bZIP* genes into a group designated as N (Fig. 2). Furthermore, gene structure analysis indicated that most members located in the same group showed similar exon/intron numbers (Fig. 3). About 74% of the *NhhbZIP* genes had no introns, which is higher than that in most higher plants, such as apple, sorghum, and soybean [4, 20, 35]. Exon/intron gain/loss plays an important role in the diversification of multiple gene families [36], and it was also observed in the present study. For instance, *NhhbZIP3* had three exons, while the paralogous gene, *NhhbZIP10*, had two, revealing a loss of an exon in the evolutionary process. A similar situation was also reported in soybean and apple bZIP families [4, 20]. These losses may derive from chromosomal rearrangements and fusions and can potentially cause the emergence of functionally distinct paralogs [37]. These lines of evidence indicate that exon/intron gain/loss might be the main driving force behind the diversity of *bZIP* genes in *Nh*. *haitanensis*. Interestingly, the lengths of exons in the genes of the same group showed significant variation in *Nh*. *haitanensis*. Several studies found that with the relatively large *bZIP* gene families in higher plants, caused by tandem or segmental duplication, some duplicated genes in the same group had nearly identical exon lengths [4, 20]. Combined with the finding that the lengths of the exons from the same group were diverse, it is therefore likely that the *Nh*. *haitanensis bZIP* gene family has not undergo tandem or segmental duplication in the evolutionary process. As expected, tandem duplication and segmental duplication events were not found in the *Nh*. *haitanensis bZIP* gene family. We hypothesized that the generation of some paralogous *NhhbZIP* genes may be caused by species-specific approaches in the evolutionary process. Additionally, motif constitution analysis showed that there were different motif constitutions among the different groups; however, most bZIP proteins in the same group harbored similar motifs (Fig. 4). For example, group N shares motifs 1 and 6, except for NhhbZIP8 and NhhbZIP18, which only contain motifs 1 and 7. Some specific motifs, such as motifs 3, 4, and 10, exist only in group I. These motifs may perform specific functions in the bZIP family of *Nh*. *haitanensis*. Further studies are clearly required to completely understand the roles of these specific motifs in *Nh*. *haitanensis*. Taken together, phylogenetic analysis of the *NhhbZIP* genes is basically congruent with the gene structures and motif compositions; there are similar exon/intron numbers and conserved motifs in every group, which has also been observed for several green plants, such as rape, grape, and *Fagopyum talaricum* [38–40].

To explore the evolutionary relationship of the *bZIP* gene family across different species, we analyzed the synteny between the *Nh*. *haitanensis bZIP* genes and their counterparts from five red algae, one brown algae, and one green algae. There were more orthologous gene pairs between *Nh*. *haitanensis* and two Bangiales algae than between *Nh*. *haitanensis* and other algae species (Fig. 6, Additional file 2: Table S2). In particular, *Nh*. *haitanensis* and *Ny*. *yezoensis* had the most orthologous gene pairs, in agreement with their closer evolutionary relationship. Within these gene pairs, 15 single collinearity gene pairs between *Nh*. *haitanensis* and *Ny*. *yezoensis* were detected, revealing that these *bZIP* genes seemed to be present in the genome of the last common ancestor of the two algae species. In addition, a number of *bZIP* genes present in *Nh*. *haitanensis* and *Po*. *umbilicalis* were not syntenic, suggesting that their genomes might have experienced multiple chromosomal rearrangements after the divergence of these two genera, in agreement with a previous study [33]. The Ka/Ks ratios for all orthologous gene pairs were far less than 1, indicating that these genes may have undergone strong purifying selection pressure during evolution.

In plants, *bZIP* genes are master regulators of many central physiological processes, including drought/osmotic stress response [7, 22, 35], pathogen defense [41, 42], and light and stress signaling [43]. However, little is known about the functions of *bZIP* genes in the regulation of the dehydration stress response in *Nh*. *haitanensis*. According to the expression data, almost all *bZIP* genes showed expression to some degree in at least one treatment, revealing that they may play an extensive role in *Nh*. *haitanensis* dehydration and rehydration. Comparing the expression data from samples both before and after dehydration/rehydration treatments, seven *bZIP* genes showed significant expression changes in response to dehydration and rehydration (Additional file 3: Table S3). Interestingly, more *bZIP* genes showed expression changes after severe dehydration and rehydration than after moderate dehydration; therefore, the bZIP family might play a more dominant role in regulating severe dehydration and rehydration. Taken together, these results suggest that *bZIP* genes contribute to strong resistance to desiccation/rehydration stress and pave the way for further research on the *bZIP*-mediated desiccation/rehydration stress response in *Nh*. *haitanensis*.

To gain insight into the function of these *bZIP* genes, gene co-expression network analysis focusing on the seven key genes was performed, followed by GO enrichment analysis of co-expression gene sets. These results indicate that the shared DEGs collectively responding to dehydration and rehydration and their corresponding network genes were mainly enriched in DNA repair, DNA metabolic process, and regulation of helicase activity. Furthermore, the two upregulated genes responding to severe dehydration and the genes in their networks were enriched in macromolecule modification, cellular catabolic process, and transmembrane transport. In addition, the three specific DEGs responding to rehydration and their co-expression gene networks were enriched in the regulation of the cell cycle process and defense response. These findings suggest that different regulatory factors and their co-expression gene networks play vital roles in specific biological functions.

## Conclusions

To summarize, we first identified and analyzed the genome-wide *bZIP* transcription factor family in *Nh. haitanensis*. Nineteen *NhhbZIP* genes were identified in the *Nh. haitanensis* genome and distributed unevenly on the 11 superscaffolds. Based on phylogenetic analysis, 19 *NhhbZIP* genes were divided into four groups, and each group had an analogous exon/intron number and motif composition, as well as diverse exon lengths. Notably, no tandem or segmental duplication events were found in the *NhhbZIP* gene family, suggesting that the evolution of *NhhbZIP* genes is not driven by tandem or segmental duplication events. Additionally, we performed a cross-species collinearity analysis of the *bZIP* family genes between *Nh. haitanensis* and seven other algae. The findings of these analyses will contribute to future comparative gene function studies. The expression profiles of *NhhbZIP* genes in response to dehydration and rehydration were displayed using the RNA-seq data. The analysis of seven key DEGs and their corresponding network genes revealed that these *NhhbZIP* genes and their co-expression gene networks are involved in diverse biological processes. This study lays a foundation for further exploring the functions of *bZIP* genes in response to dehydration and rehydration in *Nh. haitanensis* and for improving *Nh. haitanensis* in southern China.

## Methods

### Identification of *bZIP* family genes and their conserved domains in *Nh*. *haitanensis*

The high-quality *Nh*. *haitanensis* genome assemblies and protein sequences were obtained from our laboratory [30]. The known *Cy*. *merolae* and *Ga*. *sulphuraria* bZIP protein sequences were downloaded from the PlnTFDB database (http://plntfdb.bio.uni-potsdam.de/v3.0/), and *A*. *thaliana* and *Cl*. *reinhardtii* bZIP protein sequences were downloaded from the Phytozome database [44]. *Neoporphyra haitanensis bZIP* sequences were obtained through three steps. First, the Hidden Markov Model profiles of the bZIP domain (PF07716, PF03131, PF00170) obtained from the Pfam database (http://pfam.sanger.ac.uk/) were used to identify the bZIP proteins in the *Nh*. *haitanensis* dataset using HMMER software 3.0 with a threshold of e-value < e^− 5^ [45, 46]. A BLAST search was performed to identify putative bZIPs in the *Nh*. *haitanensis* database with all *Cy*. *merolae*, *Ga*. *sulphuraria*, *Cl*. *reinhardtii*, and *A*. *thaliana* bZIPs as queries. Finally, all candidate genes were examined to confirm the existence of the bZIP domain using the online program SMART (http://smart.embl-heidelberg.de/). The non-redundant and confident genes were gathered and assigned as *Nh*. *haitanensis bZIP* genes. The same process was used to obtain *Ny*. *yezoensis bZIP* family genes from its genome database [33]. Additionally, we collected sequences of the conserved domains from the identified NhhbZIP proteins. Then, the conserved domains of the NhhbZIP protein sequences were aligned using the ClustalX 1.83 program with default parameters [32]. WebLogo was used for the visualization of multiple sequence alignment of the NhhbZIP family domains [47].

### Phylogenetic analyses and classification of *NhhbZIP* family members

Two phylogenetic trees were generated, one using only NhhbZIP protein sequences and the other using the bZIP protein sequences of *Nh*. *haitanensis* and five other plant species (*Ny*. *yezoensis*, *Cy*. *merolae*, *E*. *siliculosus*, *Cl*. *reinhardtii*, and *A*. *thaliana*). *E*. *siliculosus* bZIP protein sequences were acquired from the NCBI database (https://www.ncbi.nlm.nih.gov/). Multiple alignments of protein sequences were performed by ClustalW in MEGA 7.0 with default parameters [48]. The ML method was used to construct phylogenetic trees using MEGA 7.0 [48] under the JTT + G amino acid substitution model selected based on an ML model test. The bootstrap test was carried out with 1000 replicates. The classification of all identified *NhhbZIP* family members refers to previous studies on *Arabidopsis* [8]. Finally, the phylogenetic trees were edited and visualized using the online tvBOT tool [49].

### Protein properties and sequence analyses

The CDS, MW, and other properties of the identified bZIP proteins were determined using the ExPasy website (https://web.expasy.org/). The online MEME tool (https://meme-suite.org/meme/tools/meme) was used to identify the conserved motifs in all NhhbZIP protein sequences with the following parameters: the maximum number of motifs was 10, and the optimum motif width was set between 6 and 200. The exon and intron constituents of the *NhhbZIP* members were displayed with the Gene Structure Display Server program (GSDS: http://gsds.gao-lab.org/).

### Location on superscaffolds, gene duplication of *NhhbZIP* family members, and collinearity analysis with other species

Using MapGene2Chromosome online software (http://mg2c.iask.in/mg2c_v2.0/) and *Nh*. *haitanensis* genome data [30], we visualized the location of the *bZIP* genes on superscaffolds. In addition, we investigated the tandem and segmental duplication events of the *bZIP* gene family within the *Nh*. *haitanensis* genome using BLAST and MCScanX [50] software with default parameters.

The CDSs of all genes in *Cy*. *merolae*, *Ch*. *crispus*, *Pr*. *purpureum*, *Po*. *umbilicalis*, and *E*. *siliculosus* were downloaded from the NCBI database (https://www.ncbi.nlm.nih.gov/), and those of *Ny*. *yezoensis* and *Cl*. *reinhardtii* were obtained from our laboratory and the Ensembl database (https://www.ensembl.org/index.html), respectively. The *Ch*. *crispus*, *Pr*. *purpureum*, *Po*. *umbilicalis*, and *E*. *siliculosus bZIP* genes were acquired from accessions GCA_000350225.2, GCA_008690995.1, GCA_002049455.2, and GCA_000310025.1 (NCBI), respectively. We used LAST software (https://gitlab.com/mcfrith/last) with the default parameter to perform a pairwise comparison of the CDSs of all genes between the genomes of *Nh*. *haitanensis* and seven other algae (*Cy*. *merolae*, *Ny*. *yezoensis*, *Cl*. *reinhardtii*, *Ch*. *crispus*, *Pr*. *purpureum*, *Po*. *umbilicalis*, and *E*. *siliculosus*), and then, we identified the collinearity regions from different species according to the comparison results and gene location information using JCVI software (https://github.com/tanghaibao/jcvi) with the default parameter. Diagrams were drawn using JCVI software. The Ka and Ks nucleotide substitutions between orthologous *bZIP* gene pairs were obtained using ParaAT software [51].

### Expression analysis of *NhhbZIP* genes

Using the RNA-seq data described in the previously published study [26], we explored the expression patterns of the *bZIP* genes in response to dehydration/rehydration treatments. Reads counts were normalized to the expected number of Fragments Per Kilobase of transcript sequence per Millions base pairs sequenced (FPKM). Using the DESeq2 package, DEGs were identified among various treatments (|log2(FoldChange)| ≥ 1 and adjusted *P*-value ≤ 0.05) [52]. A heatmap of the *NhhbZIP* expression profile was drawn using the pheatmap package in R.

### Algal materials and treatments

The algal material used in this study, *Nh*. *haitanensis* PH-38, was a laboratory-cultured genetically pure line, as described in our previous study [26]. Gametophytes (thalli) of this pure line were cultured in running sterilized seawater with Provasoli’s enrichment solution medium (PES) under the following conditions: 20 ± 1 °C with 50 μmol photons · m^− 2^ s^− 1^ and a 12 h:12 h light:dark (L:D) photoperiod. Before performing the experiments, the thalli were acclimatized for 2 weeks in running sterilized seawater under the following conditions: 20 ± 1 °C with 1250 μmol photons·m^− 2^ s^− 1^ and 12 h:12 h L:D photoperiod. After acclimation, the surface water was removed from the thalli with paper towels, and then, the selected thalli were naturally exposed to air at 20 ± 1 °C and 1250 μmol photons·m^− 2^ s^− 1^. The absolute water content (AWC) of the thalli was determined according to Kim et al. [53]. Algal materials under normal growth conditions were collected as the control group (AWC100). The algal samples were collected when the total water content of the algae decreased by 30% (AWC70) and 80% (AWC20). For rehydration (AWC20_REH), severely dehydrated algae (losing 80% water content) were recovered under normal conditions for 30 min [26]. Three biological replicates were used for each treatment. All samples from each treatment were immediately frozen in liquid nitrogen.

### qRT-PCR validation

Total RNA was extracted from each sample using a plant RNA extraction kit (Omega Bio-Tek, Norcross, GA, USA), and contaminating DNA was digested with RNase-Free DNase I (Tiangen, Beijing, China), following the manufacturer’s instructions. For the first-strand cDNA synthesis experiment, approximately 1 μg of purified total RNA was reverse transcribed to cDNA in a 20 μL reaction volume using a Transcriptor First Stand cDNA Synthesis Kit (Roche Molecular Biochemicals, Mannheim, BW, Germany), following the supplier’s instructions. The relative transcript levels of selected genes were analyzed by real-time PCR with the TaKaRa PCR Thermal Cycler Dice Real Time System (TaKaRa Bio Inc., Otsu, Shiga, Japan). The reactions were performed in 25 μL volumes containing 12.5 μL of 2 × TB Green Premix Ex Taq II (TaKaRa Bio Inc.), 2 μL of the diluted cDNA mix, 1 μL of each primer (0.4 μM final concentration of each primer), and 8.5 μL of RNA-free water. The qRT-PCR program was 95 °C for 30 s, followed by 40 cycles of 95 °C for 5 s and 60 °C for 30 s. To verify the specificity of each amplification reaction, the melting curves for each amplicon were also analyzed. Each PCR reaction was performed in three independent biological replicates. The primers used for qRT-PCR are listed in Additional file 5: Table S5. The ubiquitin conjugating enzyme (UBC) gene was used as an internal control [54]. The relative gene expression values were calculated using the 2^−ΔΔCt^ method [55].

### Gene co-expression networks and gene ontology enrichment analysis

Gene co-expression networks were identified using the R package WGCNA [56]. We visualized the gene co-expression network results using Cytoscape software [57]. The previously published RNA-seq data were used for gene co-expression analysis with the following parameters: the soft threshold power was set to 18, the “minModuleSize” value was set to 30, and the “mergeCutHeight” value was set to 0.25. Only genes with FPKM values > 0 in any sample were used for analysis. Pearson’s correlation coefficient was obtained using the Pearson algorithm. The co-expression networks were constructed based on all genes with a weighted correlation.

GO enrichment analysis of gene sets were conducted using TBtools [58]. We focused on biological processes. The *P*-value of each GO term was generated and adjusted using the Benjamin–Hochberg method [59].

### Electronic supplementary material

Below is the link to the electronic supplementary material.

**Additional file 1: Table S1** List of the 19 *Neoporphyra haitanensis* basic leucine zipper (*NhhbZIP*) genes identified in this study. **Table S1** List of the 19 *Neoporphyra haitanensis* basic leucine zipper (*NhhbZIP*) genes identified in this study

**Additional file 2: Table S2 (A)** Non-synonymous (Ka), synonymous (Ks), and Ka/Ks values for syntenic gene pairs in *Neoporphyra haitanensis* and *Neopyropia yezoensis*. **Table S2 (B)** Ka, Ks, and Ka/Ks values for syntenic gene pairs in *Nh. haitanensis* and *Porphyra umbilicalis*. **Table S2 (C)** Ka, Ks, and Ka/Ks values for syntenic gene pairs in *Nh. haitanensis* and *Cyanidioschyzon merolae*. **Table S2 (D)** Ka, Ks, and Ka/Ks values for syntenic gene pairs in *Nh. haitanensis* and *Ectocarpus siliculosus*. **Table S2 (E)** Ka, Ks, and Ka/Ks values for syntenic gene pairs in *Nh. haitanensis* and *Chlamydomonas reinhardtii*. **Table S2 (F)** Ka, Ks, and Ka/Ks values for syntenic gene pairs in *Nh. haitanensis* and *Chondrus crispus*. **Table S2 (G)** Ka, Ks, and Ka/Ks values for syntenic gene pairs in *Nh. haitanensis* and *Porphyridium purpureum*

**Additional file 3: Table S3 (A)** Differentially expressed genes (DEGs) in response to moderate dehydration. **Table S3 (B)** DEGs in response to severe dehydration. **Table S3 (C)** DEGs in response to rehydration

**Additional file 4: Table S4 (A)** Co-expression gene sets of the *Neoporphyra haitanensis* basic leucine zipper 6 (*NhhbZIP6*) gene. **Table S4 (B)** Co-expression gene sets of *NhhbZIP9*. **Table S4 (C)** Co-expression gene sets of *NhhbZIP10*. **Table S4 (D)** Co-expression gene sets of *NhhbZIP11*. **Table S4 (E)** Co-expression gene sets of *NhhbZIP14*. **Table S4 (F)** Co-expression gene sets of *NhhbZIP18*. **Table S4 (G)** Co-expression gene sets of *NhhbZIP19*

**Additional file 5: Table S5** Primers used in quantitative real-time polymerase chain reaction (qRT-PCR) for validating *Neoporphyra haitanensis* basic leucine zipper (*NhhbZIP*) genes

**Additional file 6: Figure S1**. Venn diagram of differentially expressed genes (DEGs) responsive to moderate dehydration (AWC70), severe dehydration (AWC20), and rehydration (AWC20_REH). This Venn diagram was drawn using the number of DEGs in the three stress treatments (AWC70, AWC20, and AWC20_REH) relative to the control group (AWC100). AWC70 *vs*. AWC100 represents DEGs between AWC70 and AWC100, AWC20 *vs*. AWC100 represents DEGs between AWC20 and AWC100, AWC20_REH *vs*. AWC100 represents DEGs between AWC20_REH and AWC100

## Data Availability

RNA-seq data of *Nh*. *haitanensis* in dehydration/rehydration treatments are available in NCBI under accession number PRJNA282903. All other datasets generated in this study are included as supplementary information of this article.

## Abbreviations

*NhhbZIP*: *Neoporphyra haitanensis bZIP*
AWC: Absolute water content
bZIP: Basic leucine zipper
CDS: Coding sequence length
DEGs: Differentially expressed genes
FPKM: Expected number of Fragments Per Kilobase of transcript sequence per Millions base pairs sequenced
GO: Gene Ontology
GSDS: Gene Structure Display Server
Ka: Non-synonymous
Ks: Synonymous
ML: Maximum Likelihood
MW: Molecular weight
PES: Provasoli’s enrichment solution medium
pI: Isoelectric point
qRT-PCR: Quantitative real-time polymerase chain reaction
REH: Rehydration
RNA-seq: RNA sequencing
UBC: Ubiquitin conjugating enzyme
WGCNA: Weighted Correlation Network Analysis
