# Supplementary material for: Characterization of the basic leucine zipper transcription factor family of Neoporphyra haitanensis and its role in acclimation to dehydration stress
Source: BMC Plant Biol. 2023 Dec 5;23:617. doi: 10.1186/s12870-023-04636-7 (PMC10696790; doi:10.1186/s12870-023-04636-7)
Supplement: Supplementary file 2 — Additional file 2: Table S2 (A) Non-synonymous (Ka), synonymous (Ks), and Ka/Ks values for syntenic gene pairs in Neoporphyra haitanensis and Neopyropia yezoensis. Table S2 (B) Ka, Ks, and Ka/Ks values for syntenic gene pairs in Nh. haitanensis and Porphyra umbilicalis. Table S2 (C) Ka, Ks, and Ka/Ks values for syntenic gene pairs in Nh. haitanensis and Cyanidioschyzon merolae. Table S2 (D) Ka, Ks, and Ka/Ks values for syntenic gene pairs in Nh. haitanensis and Ectocarpus siliculosus. Table S2 (E) Ka, Ks, and Ka/Ks values for syntenic gene pairs in Nh. haitanensis and Chlamydomonas reinhardtii. Table S2 (F) Ka, Ks, and Ka/Ks values for syntenic gene pairs in Nh. haitanensis and Chondrus crispus. Table S2 (G) Ka, Ks, and Ka/Ks values for syntenic gene pairs in Nh. haitanensis and Porphyridium purpureum [file 12870_2023_4636_MOESM2_ESM.docx]

Table S2 (A) Non-synonymous (Ka), synonymous (Ks), and Ka/Ks values for syntenic gene pairs in *Neoporphyra haitanensis* and *Neopyropia yezoensis*

| NhhbZIP Gene ID | NyybZIP Gene ID | Ka | Ks | Ka/Ks |
| --- | --- | --- | --- | --- |
| ph00473  ph00521  ph01245  ph06295  ph06500 (NhhbZIP3)  ph06775  ph06782  ph06792  ph06931  ph09590  ph09908  ph10781  ph10843  ph10936  ph11337  ph11919  ph06500 (NhhbZIP2) | py02736  py09118  py07510  py05239  py01766  py06285  py05743  py05181  py07657  py02169  py08157  py03708  py10100  py08760  py00207  py06253  py01766 | \| 0.0782854  0.0286714  0.171584  0.0745729  0.223602  0.144932  0.160606  0.0757836  0.0795492  0.084822  0.0945014  0.200061  0.10321  0.106015  0.0394168  0.135463  0.222642 \| \| --- \| | 0.976203  0.459629  0.985241  0.530452  0.803909  0.685837  0.772292  0.760153  0.885064  0.688871  0.554634  0.691189  0.697969  1.03193  0.539104  0.957088  0.808691 | 0.0801938  0.0623795  0.174154  0.140584  0.278143  0.211322  0.207961  0.0996951  0.0898796  0.123132  0.170385  0.289444  0.147872  0.102735  0.0731153  0.141537  0.275311 |

NhhbZIP indicates *Nh*. *haitanensis* basic leucine zipper; NyybZIP indicates *Ny*. *yezoensis* basic leucine zipper

Table S2 (B) Ka, Ks, and Ka/Ks values for syntenic gene pairs in *Nh*. *haitanensis* and *Porphyra umbilicalis*

| NhhbZIP Gene ID | PoubZIP Gene ID | Ka | Ks | Ka/Ks |
| --- | --- | --- | --- | --- |
| ph00521  ph01245  ph06082  ph06295  ph06775  ph06931  ph09908  ph10843  ph10936 | BU14_0023s0052  BU14_0513s0012  BU14_0244s0008  BU14_0014s0072  BU14_0033s0070  BU14_0086s0015  BU14_0183s0032  BU14_0072s0093  BU14_0216s0011 | \| 0.20041  0.60677  0.37596  0.26999  0.34555  0.14932  0.12960  0.29053  0.26996 \| \| --- \| | 3.22261  2.00221  2.87209  2.74959  2.28798  1.53252  1.93987  3.41881  3.6942 | 0.062191  0.303055  0.130904  0.098196  0.151031  0.097438  0.066813  0.084981  0.073079 |

NhhbZIP indicates *Nh*. *haitanensis* basic leucine zipper; PoubZIP indicates *Po*. *umbilicalis* basic leucine zipper

Table S2 (C) Ka, Ks, and Ka/Ks values for syntenic gene pairs in *Nh*. *haitanensis* and *Cyanidioschyzon merolae*

| NhhbZIP Gene ID | CymbZIP Gene ID | Ka | | Ks | Ka/Ks |
| --- | --- | --- | --- | --- | --- |
| NO |  | \| \| 9 \| \| --- \| \| \| --- \| --- \| | \|  \| \| --- \| | | \|  \| \| --- \| |

NhhbZIP indicates *Nh*. *haitanensis* basic leucine zipper; CymbZIP indicates *Cy*. *merolae* basic leucine zipper

Table S2 (D) Ka, Ks, and Ka/Ks values for syntenic gene pairs in *Nh*. *haitanensis* and *Ectocarpus siliculosus*

| NhhbZIP Gene ID | EsbZIP Gene ID | Ka | | Ks | Ka/Ks |
| --- | --- | --- | --- | --- | --- |
| NO |  | \| \| 9 \| \| --- \| \| \| --- \| --- \| | \|  \| \| --- \| | | \|  \| \| --- \| |

NhhbZIP indicates *Nh*. *haitanensis* basic leucine zipper; EsbZIP indicates *E*. *siliculosus* basic leucine zipper

Table S2 (E) Ka, Ks, and Ka/Ks values for syntenic gene pairs in *Nh*. *haitanensis* and *Chlamydomonas reinhardtii*

| NhhbZIP Gene ID | ClrbZIP Gene ID | Ka | | Ks | Ka/Ks | |
| --- | --- | --- | --- | --- | --- | --- |
| NO |  | \| \| 9 \| \| --- \| \| \| --- \| --- \| | \|  \| \| --- \| | |  |  |

NhhbZIP indicates *Nh*. *haitanensis* basic leucine zipper; ClrbZIP indicates *Cl*. *reinhardtii* basic leucine zipper

Table S2 (F) Ka, Ks, and Ka/Ks values for syntenic gene pairs in *Nh*. *haitanensis* and *Chondrus crispus*

| NhhbZIP Gene ID | ChcbZIP Gene ID | Ka | | Ks | Ka/Ks |
| --- | --- | --- | --- | --- | --- |
| NO |  | \| \| 9 \| \| --- \| \| \| --- \| --- \| | \|  \| \| --- \| | | \|  \| \| --- \| |

NhhbZIP indicates *Nh*. *haitanensis* basic leucine zipper; ChcbZIP indicates *Ch*. *crispus* basic leucine zipper

Table S2 (G) Ka, Ks, and Ka/Ks values for syntenic gene pairs in *Nh*. *haitanensis* and *Porphyridium purpureum*

| NhhbZIP Gene ID | PrpbZIP Gene ID | Ka | | Ks | Ka/Ks |
| --- | --- | --- | --- | --- | --- |
| NO |  | \| \| 9 \| \| --- \| \| \| --- \| --- \| | \|  \| \| --- \| | | \|  \| \| --- \| |

NhhbZIP indicates *Nh*. *haitanensis* basic leucine zipper; PrpbZIP indicates *Pr*. *purpureum* basic leucine zipper
