# Supplementary material for: Characterization of the basic leucine zipper transcription factor family of Neoporphyra haitanensis and its role in acclimation to dehydration stress
Source: BMC Plant Biol. 2023 Dec 5;23:617. doi: 10.1186/s12870-023-04636-7 (PMC10696790; doi:10.1186/s12870-023-04636-7)
Supplement: Supplementary file 5 — Additional file 5: Table S5 Primers used in quantitative real-time polymerase chain reaction (qRT-PCR) for validating Neoporphyra haitanensis basic leucine zipper (NhhbZIP) genes [file 12870_2023_4636_MOESM5_ESM.docx]

**Table S5** Primers used in quantitative real-time polymerase chain reaction (qRT-PCR) for validating *Neoporphyra haitanensis* basic leucine zipper (*NhhbZIP*) genes

| Gene | Primer sequences (5'→3') forward/reverse | Amplicon size (bp) |
| --- | --- | --- |
| *NhhbZIP1* | CACGTCCTCCCAGACAGACT/  GGCGGCTTTTGCTGGTT | 88 |
| *NhhbZIP5* | CCGTGATGTCGCCTGTTTT/  CACAGCCTCGTCTGCTTTCTT | 125 |
| *NhhbZIP6* | TGTCGTCTCCATCCCTATTCAC/ AGAGCAGAAACCCCGCATC | 117 |
| *NhhbZIP10* | GGGGATGTGTTGTTGTGGTTT/  ATCAAGGCATGGGACTGGTT | 90 |
| *NhhbZIP11* | GCTGTCCTCTTCTCTTGAGTTCGT/  TCTTTTGTGGTGCCTTGTGG | 140 |
| *NhhbZIP18* | CATTACTGTTGGCTGACCCTGA/  ACGCAAACGCACTCGCTAC | 99 |
| *UBC* | TCACAACGAGGATTTACCACC/  GAGGAGCACCTTGGAAACG | 107 |
