# Supplementary material for: Characterization of the basic leucine zipper transcription factor family of Neoporphyra haitanensis and its role in acclimation to dehydration stress
Source: BMC Plant Biol. 2023 Dec 5;23:617. doi: 10.1186/s12870-023-04636-7 (PMC10696790; doi:10.1186/s12870-023-04636-7)
Supplement: Supplementary file 6 — Additional file 6: Figure S1. Venn diagram of differentially expressed genes (DEGs) responsive to moderate dehydration (AWC70), severe dehydration (AWC20), and rehydration (AWC20_REH). This Venn diagram was drawn using the number of DEGs in the three stress treatments (AWC70, AWC20, and AWC20_REH) relative to the control group (AWC100). AWC70 vs. AWC100 represents DEGs between AWC70 and AWC100, AWC20 vs. AWC100 represents DEGs between AWC20 and AWC100, AWC20_REH vs. AWC100 represents DEGs between AWC20_REH and AWC100 [file 12870_2023_4636_MOESM6_ESM.docx]

**2**

**2**

**0**

**3**

**0**

**0**

**0**

AWC70 *vs*. AWC100

AWC20 *vs*. AWC100

AWC20_REH *vs*. AWC100

**Figure S1.** Venn diagram of differentially expressed genes (DEGs) responsive to moderate dehydration (AWC70), severe dehydration (AWC20), and rehydration (AWC20_REH). This Venn diagram was drawn using the number of DEGs in the three stress treatments (AWC70, AWC20, and AWC20_REH) relative to the control group (AWC100). AWC70 *vs*. AWC100 represents DEGs between AWC70 and AWC100, AWC20 *vs*. AWC100 represents DEGs between AWC20 and AWC100, AWC20_REH *vs*. AWC100 represents DEGs between AWC20_REH and AWC100.
